# Rethinking Neonatal Body Proportions: Upper Segment to Lower Segment Ratios in a Rural South Indian Cohort

**DOI:** 10.7759/cureus.104193

**Published:** 2026-02-24

**Authors:** Vinitha Arjula, Manohar Bekkam, Nikitha Pasam, Laxman Sanepu, Preethi Subramanian, Sudharshanraj Chitgupikar

**Affiliations:** 1 Pediatrics, Prasad Hospital - Children and Multi-Specialty Hospital, Kukatpally, Hyderabad, IND; 2 Pediatrics, MediCiti Institute of Medical Sciences, Hyderabad, IND

**Keywords:** anthropometry, body proportions, cross-sectional studies, crown-heel length, neonatal growth, reference values

## Abstract

Introduction

The upper segment to lower segment (US:LS) ratio is a key anthropometric measure for assessing body proportions in children, which provides insight into disorders with disproportionate growth. However, body proportions are governed by ethnic, genetic, nutritional, and environmental factors. This study aimed to establish US:LS ratio reference values in live-born neonates from rural Telangana, India; compare US:LS ratios across sex, gestational age categories, birth weight categories, and intrauterine growth status; and assess whether Western normative values apply to this population.

Methods

A cross-sectional study was conducted between January and December 2021, at MediCiti Institute of Medical Sciences, Hyderabad, Telangana, India, and 998 neonates were enrolled. Crown-heel length was measured with an infantometer, and the lower segment from pubic symphysis to heel with the help of an inch tape, and the upper segment was derived by subtraction. Neonates were classified by sex, gestational age (preterm, term, post-term), birth weight categories, and intrauterine growth status, and their US:LS ratio was compared. Descriptive statistics included mean and standard deviation for continuous variables and percentages for categorical variables. Independent t-tests were used to compare means between two groups. One-way ANOVA with post-hoc Tukey’s honestly significant difference (HSD) was used for comparing more than two groups. A p-value of <0.05 was considered statistically significant.

Results

The study included 998 neonates, of whom 530 (53.1%) were males, and 468 (46.9%) were females. The mean gestational age ± SD was 38.16 ± 1.29 weeks. The overall mean US:LS ratio was 1.511 ± 0.13. Male neonates exhibited a significantly higher ratio than females (1.519 ± 0.14 vs. 1.502 ± 0.13; p = 0.048). Preterm neonates had a significantly higher mean ratio compared to term infants (1.56 ± 0.15 vs. 1.510 ± 0.13; p = 0.0031). Among gestational age groups, only the comparison between < 32 weeks and 37-39 weeks reached significance (p = 0.0083).

Conclusion

The mean US:LS ratio of neonates is lower than the typical values available in the literature. There is a gender difference observed with male neonates having a greater US:LS ratio. The mean US:LS ratio differs between preterm and term neonates. There is a need for further large-scale multicentric population-level studies on the US:LS ratio among Indian children. Clinicians should consider population-specific reference values when evaluating neonates for suspected skeletal dysplasia or disproportionate growth.

## Introduction

Fetal growth is driven predominantly by insulin and insulin-like growth factors (IGF-I, IGF-II), with growth hormone having a limited effect during this period. After birth, growth velocity declines, particularly in the first year of life, from 25 cm/year at birth to approximately 10 cm/year by the end of infancy [1]. Growth in this phase is influenced primarily by nutrition and thyroid hormone. As the child grows through later stages of childhood and puberty, the primary drivers of growth become growth hormone, IGF-1, thyroid hormone, and sex steroids [2]. In addition to linear growth, body proportions also undergo substantial changes. At birth, the lower limbs contribute around 30% to standing height, increasing to about 48% by the time of puberty [3].

The upper segment to lower segment (US:LS) ratio is an important anthropometric parameter used to assess body proportions and growth symmetry, especially in the evaluation of short stature and skeletal dysplasia [4]. At birth, normative references from the Western literature consistently cite a US:LS ratio of approximately 1.7, with a gradual decline to 1.0 by seven to 10 years of age due to differential growth in limbs and trunk [5-8]. During the first five years, the upper and lower segments grow at similar rates. Between five years and puberty, the lower segment contributes more significantly to height, and the US:LS ratio progressively declines [4]. At puberty, a reversal occurs due to rapid limb growth, followed by stabilization post-maturity.

However, recent studies in different populations have challenged these benchmarks. A study by Tanaka et al. [9] among children of Japan in the age group of 5.5 -17.5 years provided different US:LS ratios for boys and girls and also plotted percentiles for the same, which defies the standard norm of 1.7. While there have been no gender differences noted in US:LS ratio in the literature, Turan S et al. [10] published a study on the US:LS ratio of three- to 18-year-old school children and found that the mean US:LS in boys was more than that of girls across these age groups. Sandhya et al. [11] reported that among 333 boys and 209 girls in the age group of zero to one year of their study population, at birth (zero year), girls had a mean US:LS ratio of 1.44, and boys at birth had 1.45.

Despite the clinical relevance of the US:LS ratio, to date, only one Indian study by Sandhya et al. [11] has reported US:LS ratios across age groups, including infants. The currently accepted US:LS ratios are derived from Western populations and may not accurately represent Indian neonates. This study aimed to establish US:LS ratio reference values in live-born neonates from rural Telangana, India; compare US:LS ratios across sex, gestational age categories, birth weight categories, and intrauterine growth status; and assess whether Western normative values are applicable to this population. 

## Materials and methods

A cross-sectional study was conducted at MediCiti Institute of Medical Sciences, Hyderabad, Telangana, between January 1, 2021, and December 31, 2021, after approval from the Institutional Ethics Committee (EC/18/XII/2K20(5/6) Revised). All live-born neonates delivered during this period were included in the study. A total of 998 neonates were included after applying the exclusion criteria.

Inclusion criteria

All live-born neonates delivered at the institution during the study period.

Exclusion criteria

The exclusion criteria for this study comprised multiple pregnancies, such as twins and triplets, as well as neonates presenting with gross congenital skeletal anomalies. In addition, neonates exhibiting syndromic features, especially anomalies involving limbs and face, alongside stillbirths and intrauterine demises (IUD), were not included in the analysis.

Informed written consent was obtained from the parents of all neonates included in the study. A pre-formatted proforma was used to collect the relevant data on neonates, such as date and time of birth, birth weight, head circumference, length, and upper segment lower segment ratio (US:LS), along with general examination. A single senior paediatric resident who was already trained in measuring anthropometry for two years was involved for the measurement of anthropometrical indices. All the measurements were taken thrice, at 48 hours of life, and the average of the findings was noted to minimize errors. Birth weight was recorded after stabilizing the neonate. The weight was measured on a digital weighing machine with a variability of ± 10 grams. Recumbent length was measured using an infantometer; the lower segment was measured from the pubic symphysis to the heel using a non-stretchable tape. The upper segment was derived by subtracting the lower segment from the total length, and the US:LS ratio was arrived at. Head circumference was measured using standard methods. The birth weight of all the neonates was plotted on Fenton 2013 [12] for preterm neonates and INTERGROWTH-21 [13] for term neonates. As an institutional policy, all antenatal mothers underwent a dating scan in the first trimester. Thus, first trimester dating scan was used to calculate and confirm the gestational age. Based on gestational age, birth weight, and sex, neonates were classified as appropriate for gestational age (AGA), small for gestational age (SGA), and large for gestational age (LGA).

Statistical analysis

Data were entered in Microsoft Excel (Microsoft Corp., USA) and analysed using IBM SPSS Statistics for Windows, Version 22.0 (released 2013, IBM Corp., Armonk, NY) and Jamovi 2.6.26 (The jamovi project, 2025, retrieved from https://www.jamovi.org, Sydney, Australia). Descriptive statistics included mean and standard deviation for continuous variables and percentages for categorical variables. Independent t-tests were used to compare means between two groups. One-way ANOVA with post-hoc Tukey’s HSD was used for comparing more than two groups. A p-value of <0.05 was considered statistically significant. Cohen’s D was used to assess the effect size.

## Results

Among 1,029 live-born neonates delivered during the study period, 998 neonates were included in the study, based on our inclusion and exclusion criteria (twin neonates: 22; skeletal dysplasia: 2; syndromic neonate: 1; congenital anomalies involving the face and limbs: 6).

The study included 998 neonates, of whom 530 (53.1%) were males and 468 (46.9%) were females. A total of 65 (11.8%) neonates were preterm, and 932 (88.2%) were term. Among all the neonates, 743 (74.5%) were AGA, 18 (1.8%) were LGA, and 237 (23.7) were SGA. The mean gestational age ± SD was 38.16 ± 1.29 weeks. The mean birth weight ± standard deviation (SD) was 2840 ± 425.4 grams. The mean length ± SD was 48.29 ± 1.89 cm.

The mean US:LS ratio (and its standard deviation) among all neonates was 1.511 ± 0.13 (95% CI: 1.503-1.519). The mean US:LS ratio (and its standard deviation) for male neonates was 1.519 ± 0.14 (95% CI: 1.507 to 1.531). Meanwhile, that of female neonates was 1.502 ± 0.13 (95% CI: 1.489 to 1.515). The difference between the mean US:LS ratio among male and female neonates was calculated by an independent t-test. The p-value was 0.0481, which was statistically significant. The mean difference between sexes was 0.017 (95% CI: −0.0004 to 0.0344), corresponding to a very small effect size (Cohen’s d = 0.12). Although statistically significant, the magnitude of difference was small (Cohen’s d = 0.12), suggesting limited clinical relevance.

**Figure 1:**
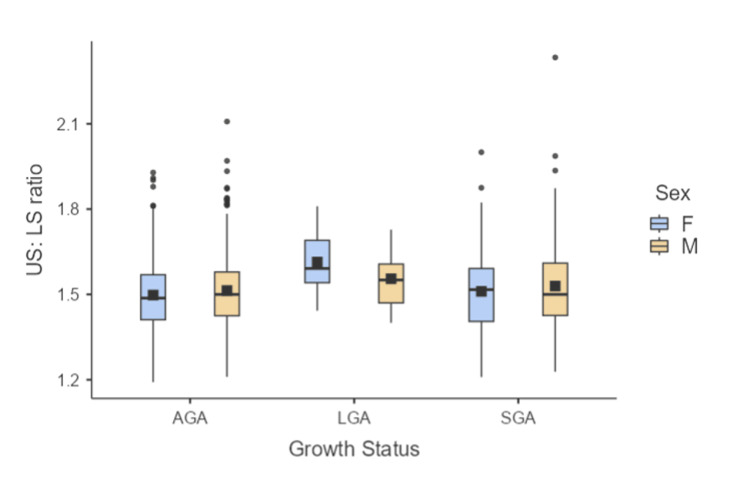
Box-whisker plot showing the distribution of US:LS ratio of neonates across intrauterine growth statuses US:LS: upper segment to lower segment; AGA: appropriate for gestational age; LGA: large for gestational age; SGA: small for gestational age, F: female; M: male

Among all the neonates, 743 were appropriate for gestational age (373 male and 370 female), 18 were large for gestational age (12 male and six female), and 237 were small for gestational age (145 male and 92 female). The comparison of the US:LS ratio of the neonates across different intrauterine growth status at birth is shown in Table 1 and Figure 1. The mean US:LS ratio was not significantly different across the different intrauterine growth statuses.

**Table 1:** Comparison of mean(+/-SD) of US:LS ratio across neonates of different intrauterine growth statuses at birth AGA: appropriate for gestational age; LGA: large for gestational age; SGA: small for gestational age; SD: standard deviation

Intrauterine growth status	Total number of neonates (n)	Mean ±SD US:LS ratio	Number of males	Mean ±SD US:LS ratio of males	Number of females	Mean ±SD of US:LS ratio of females	Statistical analysis by ANOVA and post-hoc
AGA	743	1.51 ±0.13	373	1.51 ±0.13	370	1.50 ±0.12	p = 0.0625
LGA	18	1.57 ±0.11	12	1.56 ±0.1	6	1.61±0.12	
SGA	237	1.52 ±0.15	145	1.53 ±0.16	92	1.51±0.15	
Total	998	1.511 ± 0.13	530	1.519 ± 0.14	468	1.502 ± 0.13	0.0481

Among the total number of neonates (n = 998), 932 were term neonates (497 males and 435 females), 65 were preterm (33 males and 32 females), and one was a post-term neonate (female). The mean US:LS ratio for preterm (<37 weeks; n = 65) was 1.56±0.15 (95% CI: 1.523-1.597), and for term (37-42 weeks; n = 932), it was 1.510 ± 0.13 (95% CI: 1.502-1.518), and for post-term (n = 1), it was 1.50. The difference between the mean US:LS ratio of preterm and term neonates was statistically significant with a p-value of 0.0031. The mean difference was 0.05 (95% CI: 0.013-0.087), corresponding to a small-to-moderate effect size (Cohen’s d = 0.38).

**Figure 2:**
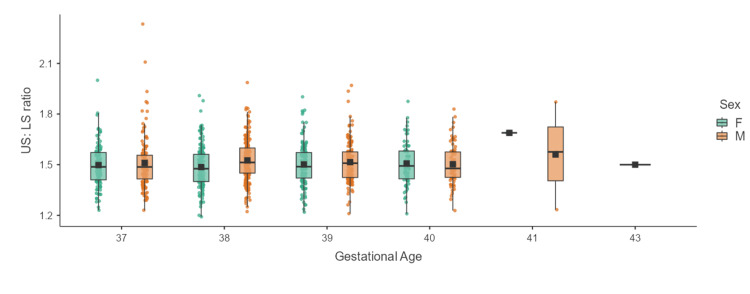
Box-whisker plot showing the distribution of US:LS ratios of neonates across gestational ages of 37-43 weeks US:LS: upper segment to lower segment; F: females; M: males

**Figure 3:**
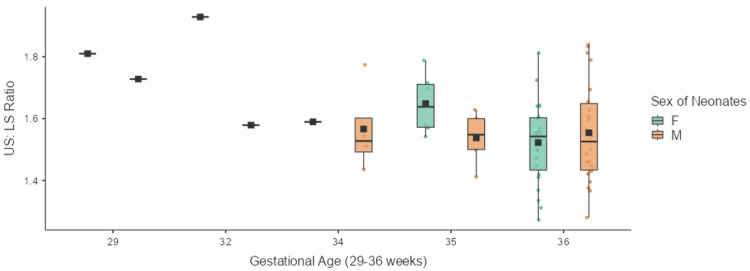
Box-whisker plot showing the distribution of US:LS ratios of neonates across gestational ages of 29-36 weeks US:LS: upper segment to lower segment; F: females; M: males

Table 2 shows the mean and standard deviation of US:LS ratios across various gestational ages. The difference was statistically significant with a p-value of 0.0083. Post-hoc test revealed a significant difference between the <32 weeks category and the 37-39 weeks category. All other comparisons across other gestational categories failed to gain significance. Figures 2-3 show the box-whisker plots representing the distribution of US:LS among term and preterm neonates across various gestational weeks.

**Table 2:** Comparison of the mean(+/-SD) of US:LS ratio across neonates of different gestational ages SD: standard deviation; US:LS: upper segment to lower segment *Post-hoc test revealed significant difference between the <32 weeks category and 37-39 weeks category.

Gestational age categories (weeks)	Number of neonates (n)	Mean (±SD) of the US:LS ratio	Statistical analysis by ANOVA and Tukey’s HSD post-hoc analysis*.
<32 weeks	2	1.77 (±0.04)	p = 0.0083
32-33 weeks	2	1.62 (±0.18)	
34-36 weeks	61	1.55 (±0.14)	
37-39 weeks	789	1.51 (±0.13)	
40-42 weeks	143	1.51 (±0.13)	
>42 weeks	1	1.50 (±0.00)	

The mean US:LS ratios of term and late preterm neonates were compared. The mean US:LS ratio of term neonates (≥37-42 weeks; n = 932) was 1.51 ± 0.13 (95% CI: 1.502-1.518). The mean US:LS ratio of late preterm neonates (34-37 weeks; n = 61) was 1.55 ± 0.14 (95% CI: 1.515-1.585). The difference between the means of US:LS ratios between late preterm and term neonates was statistically significant (p = 0.0207). The mean difference was 0.04 (95% CI: 0.004-0.076), corresponding to a small effect size (Cohen’s d = 0.31).

**Figure 4:**
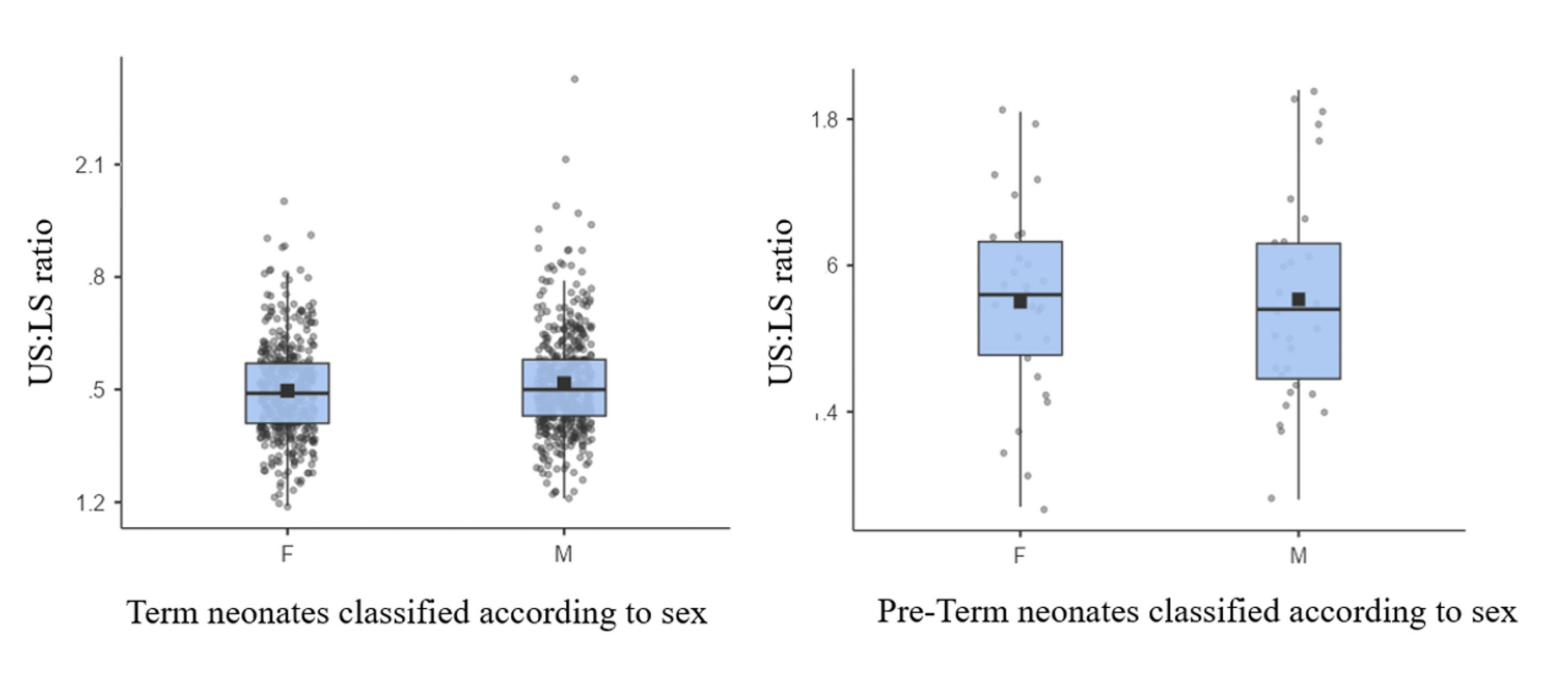
Box-whisker plot showing the distribution of US:LS ratio of male and female neonates across term and late preterm categories US:LS: upper segment to lower segment; F: female ; M: male

The mean US:LS ratio of male term neonates was 1.52 ± 0.135 and of preterm male neonates was 1.55 ± 0.15. The difference between the mean US:LS ratios of male neonates across term and late preterm categories was not statistically significant (p = 0.2336). The mean US:LS ratio of female term neonates was 1.50 ± 0.128, and that of late preterm female neonates was 1.55 ± 0.13. The difference between the mean US:LS ratios of female neonates across term and late preterm categories was statistically significant (p = 0.0498). Tables 3-4 and Figure 4 show the data tabulated and in box-whisker plots, respectively, representing the distribution of US:LS among male and female neonates in term and late preterm categories.

**Table 3:** Comparison of the mean(+/-SD) of US:LS ratios across late preterm and term neonates SD: standard deviation; US:LS: upper segment to lower segment

Gestational age	Number of neonates (n)	Mean (±SD) of US:LS ratio	Statistical analysis
Term (≥37-42 weeks)	932	1.51 ± 0.13	p = 0.0207
Late preterm (≥34 weeks to <37 weeks)	61	1.55 ± 0.14	

**Table 4:** Comparison of the mean(+/-SD) of US:LS ratio across late preterm and term neonates across genders SD: standard deviation; US:LS: upper segment to lower segment

Sex of neonates (n)	Gestational age	Mean (±SD) of US:LS ratio	Statistical analysis
Males (530)	Term (≥37-42 weeks)	1.52 ± 0.135	p = 0.2336
	Late preterm (≥34 weeks to <37 weeks)	1.55 ± 0.15	
Females (465)	Term (≥37-42 weeks)	1.50 ± 0.128	p = 0.0498
	Late preterm (≥34 weeks to <37 weeks)	1.55 ± 0.13	

**Figure 5:**
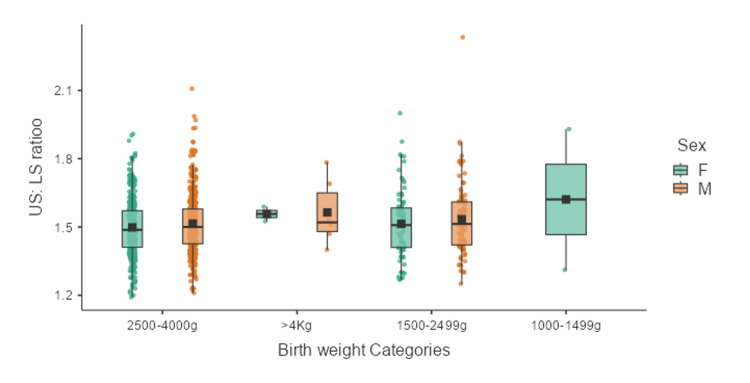
Box-whisker plot showing the distribution of US:LS ratio of neonates across birth weight categories US:LS: upper segment to lower segment; F: females; M: males

The distribution of neonates based on birth weight categories is provided in Table 5 and Figure 5. The mean US:LS across various birth weight categories was not significantly different (p = 0.369).

**Table 5:** Comparison of the mean(+/-SD) of US:LS ratio across neonates of different birth weight categories SD: standard deviation; US:LS: upper segment to lower segment

Birth weight in grams	Number of neonates (N)	Mean (±SD) of US:LS ratio	Statistical analysis by ANOVA and and Tukey’s HSD post-hoc analysis.
1000-1499	2	1.62 (±0.44)	p = 0.369
1500-2499	176	1.52 (±0.15)	
2500-4000	812	1.51 (±0.13)	
Above 4000	8	1.56 (±0.12)	

## Discussion

The present study compiles the data for US:LS ratios of neonates from the rural Telangana population and analyzes the differences across genders, different birth weight categories, gestational ages, and different intrauterine growth rates. The mean (±SD) of the US:LS ratio (1.511 ± 0.13) was observed to be lower than the traditionally cited textbook values of 1.7, which are largely derived from the Western population. The mean (±SD) of US:LS was significantly higher in males than in females at birth, but the size effect showed reduced clinical relevance. The mean (±SD) of the US:LS ratio significantly varies according to gestational ages.

The mean US:LS ratio at birth has been accepted as 1.7 by multiple authors/standard textbooks in the paediatric literature over multiple decades [5,6,14,15]. The mean US:LS ratio at birth observed in this study (1.51) is similar to a cross-sectional study done by Sandhya et al. [11]. In their study, the authors observed the US:LS ratio of children (n = 5,454) from Pune in Western India across one to 18 years and both genders. Among the 5,454 children, they studied the US:LS ratio of 333 boys and 209 girls in the age group of zero to one year. The authors observed that at birth (zero year), girls had a mean US:LS ratio of 1.44, and boys at birth had 1.45.

Our findings are corroborated by recent data from Chellappan et al. [16], who studied 408 term neonates from Southern India (Tamil Nadu), measuring lower limb length from the greater trochanter to the lateral malleolus. Calculating the US:LS ratios from their published data yielded mean ratios of 1.49 for the US:LS ratio. These values closely align with our findings despite the different lower limb measurement techniques. The concordance in both the overall ratios and the sex-based difference (boys > girls) across different regions of South India strengthens the evidence that US:LS ratios in Indian neonates are substantially lower than Western references.

In the present study, the mean US:LS ratio at birth for boys was 1.519 (±0.14) and for girls 1.502 (±0.13), which was statistically significant. In the study by Sandhya et al. [11], no significant difference was observed across genders. Chellapan et al. [16] measured lower limb length among 195 boys and 213 girls at a gestational age of 37-40 weeks. The US:LS ratio derived from the data shows a mean value of 1.50 for boys and 1.48 for girls. Turan S et al. [10] published a study on the US:LS ratio of three-18-year-old school children and found that the mean US:LS in boys was more than that of girls across these age groups. However, the statistical comparison between the sexes was not available. Although it is comparable and could be extrapolated to what is observed in the present study, the age group of neonates was not part of their study sample. The available studies in the literature are tabulated in Table 6.

**Table 6:** US:LS of children - available data from the literature US:LS: upper segment to lower segment

S. No.	Country/region of study	Authors	Year of publication	Age range of the study population	US:LS ratio - males	US:LS ratio - females
1	USA (North American) [17]	Wilkins L	1966	4 years to maturity	1.24 at 4 years declining to 0.95 at maturity	1.22 at 4 years remaining around 1.0
2	Turkey [10]	Turan S et al.	2005	3-18 years	1.108 at 3 years, nadir 0.922 at 15 years	1.098 at 3 years, nadir 0.946 at 13 years
3	Japan [9]	Tanaka C et al.	2004	5.5-17.5 years	Declining from 5.5 years, lowest (1.13) at 13.5 years	Declining from 5.5 years, lowest (1.18) at 11.5 years
4	India (Pune, Western Maharashtra) [11]	Sandhya (Kondpalle S) et al.	2019	Birth to 18 years	1.45 at birth, 1.35 at 1 year, nadir 0.89 at 14 years	1.44 at birth, 1.35 at 1 year, nadir 0.89 at 12 years
5	India (South-Tamil Nadu) [16]	Chellappan et al.	2024	Neonates (37-40 weeks)	1.46-1.52	1.35-1.53
6	India (Rural Telangana) - present study	Current study		Birth (neonates only)	1.519 ± 0.14 at birth	1.502 ± 0.13 at birth

The mean US:LS ratio for preterm (<37 weeks; n = 65) was 1.56 ± 0.15; term (37-42 weeks; n = 932) was 1.510 ± 0.1323, and post-term (n = 1) was 1.50. The difference between the US:LS ratio of preterm and term neonates was statistically significant. It showed that preterm had a higher mean US:LS ratio. This could be because much of the length (crown-heel length) of a fetus is contributed by the crown rump length [18,19] as the long bones, which contribute to the lower segment of a fetus grows slowly and in later gestational ages as the fetus matures. This observation aligns with established principles of fetal growth, where long bone growth accelerates in the later weeks of pregnancy.

The difference in the US:LS ratio between late preterm (≥34 to <37 completed GA) neonates and term neonates was also statistically significant. This difference was seen among female neonates when compared in the above categories, but the US:LS ratio was not significantly different between males of late preterm and term neonates. The statistical difference observed between females of late preterm and term neonates is clinically less relevant, as the study is not sufficiently powered for extrapolating the results to all late preterm female neonates.

The mean US:LS ratio across various intrauterine growth status and birthweight categories did not have a significant difference as noted above. There is not much data for comparison in the literature on these points.

In the present study, it was observed that the US:LS ratio did not vary significantly across different birth weight categories or intrauterine growth status (AGA, SGA, and LGA), suggesting that body proportions remain relatively constant despite variations in overall size. This finding may indicate that growth restriction or macrosomia affects the upper and lower segments proportionally, maintaining similar body proportions across the growth spectrum.

The findings of this study have important implications for clinical practice in India. The mean US:LS ratio of 1.51 at birth in our population is significantly lower than the conventional Western reference value of 1.7 that is currently cited in standard pediatric textbooks [5,6,14,15]. Clinicians evaluating Indian neonates for suspected skeletal dysplasia or disproportionate growth should consider these population-specific values when interpreting body proportions. Data from this study guides us towards a US:LS ratio between 1.38 and 1.64 (mean ± 2SD) for neonates from rural Telangana and possibly similar Indian populations, rather than the Western standard of approximately 1.7, which needs to be supported from large-scale data of neonates across the Indian population from both rural and urban origins for better generalization of results.

Although a statistically significant difference was observed between male and female neonates, the effect size was very small (Cohen’s d = 0.12), suggesting limited clinical relevance. However, further large-scale studies could clarify this.

Limitations

The sample size was small and had a lesser representation of preterm neonates. This restricts the generalizability of results. In the present study, the upper segment was calculated indirectly by subtracting the lower segment from the crown-heel length, rather than measuring sitting height directly. While this indirect method is practical and widely used in neonatal anthropometry, it may introduce cumulative measurement error from both the crown-heel length and lower segment measurements. However, in neonates, any such discrepancies are minimized as body composition variations are less pronounced compared to older children. Though a single resident was involved for all anthropometric measurements, intraobserver reliability was not separately tested. However, measuring the anthropometric indices thrice and averaging would help reduce this to some extent.

Future directions

The development of comprehensive percentile curves or nomograms specific to Indian newborns represents an important next step. The construction of percentile curves requires much larger sample sizes with adequate representation across all subgroups, particularly preterm neonates, who were underrepresented in our study. In addition, longitudinal data tracking body proportion changes from birth through childhood and adolescence would provide valuable insights into normal growth patterns in Indian children and help establish age- and sex-specific diagnostic cutoffs.

## Conclusions

The mean US:LS ratio of neonates is lower than the typical values available in the literature. The mean US:LS ratio differs between preterm and term neonates. There is a need for further large-scale multicentric population-level studies on the US:LS ratio among Indian children. Clinicians should consider population-specific reference values when evaluating neonates for suspected skeletal dysplasia or disproportionate growth.
